# Consistent Healthcare Safety Recommendation System for Preventing Contagious Disease Infections in Human Crowds

**DOI:** 10.3390/s23239394

**Published:** 2023-11-24

**Authors:** Mohammed Amoon, Torki Altameem, Mohammed Hashem

**Affiliations:** 1 Department of Computer Science, Community College, King Saud University, P.O. Box 28095, Riyadh 11437, Saudi Arabia; altameem@ksu.edu.sa; 2 Department of Dental Health, College of Applied Medical Sciences, King Saud University, P.O. Box 12372, Riyadh 12372, Saudi Arabia; mihashem@ksu.edu.sa

**Keywords:** contagious disease, personal healthcare, random forest, wearable sensor

## Abstract

The recent impact of COVID-19, as a contagious disease, led researchers to focus on designing and fabricating personal healthcare devices and systems. With the help of wearable sensors, sensing and communication technologies, and recommendation modules, personal healthcare systems were designed for ease of use. More specifically, personal healthcare systems were designed to provide recommendations for maintaining a safe distance and avoiding contagious disease spread after the COVID-19 pandemic. The personal recommendations are analyzed based on the wearable sensor signals and their consistency in sensing. This consistency varies with human movements or other activities that hike/cease the sensor values abruptly for a short period. Therefore, a consistency-focused recommendation system (CRS) for personal healthcare (PH) was designed in this research. The hardware sensing intervals for the system are calibrated per the conventional specifications from which abrupt changes can be observed. The changes are analyzed for their saturation and fluctuations observed from neighbors within the threshold distance. The saturation and fluctuation classifications are performed using random forest learning to differentiate the above data from the previously sensed healthy data. In this process, the saturated data and consistency data provide safety recommendations for the moving user. The consistency is verified for a series of intervals for the fluctuating sensed data. This alerts the user if the threshold distance for a contagious disease is violated. The proposed system was validated using a prototype model and experimental analysis through false rates, data analysis rates, and fluctuations.

## 1. Background

Wearable sensor devices are commonly used to capture healthcare details via an Internet connection. Wearable sensor devices are used for people’s health monitoring systems. A wearable sensor device-based monitoring system was used during the COVID-19 pandemic. The sensor devices measure the symptoms and conditions of people in public crowds [1,2]. Wearable devices provide healthcare information for analysis and detection processes, and use a remote monitoring system that analyzes the health condition of infected patients in public places [3]. The wireless sensor produces optimal data for monitoring and detection, ensuring people’s safety in public crowds [4]. Highly sensitive wearable sensor devices are also used for healthcare monitoring in public places. The wearable sensor devices gather the health condition data of patients via an Internet connection [5]. A body temperature sensor (BTS) is implemented in the device to detect people’s exact body temperature levels. Wearable sensor devices reduce health monitoring systems’ time and energy consumption ratios in public places [6].

Consistent wearable sensor devices are used for recommendations and precise health condition detection processes. The wearable sensor gathers the information via mobile connectivity [7]. A data analysis technique analyzes the information and produces the necessary details for the detection process. Machine learning (ML)-based tools are used to analyze the data for detection [8]. ML tools track healthcare problems based on the condition of patients. ML tools identify infectious diseases and symptoms that improve the accuracy of health condition detection [9]. The wearable sensor devices collect complex healthcare data regularly, reducing the detection latency. The ML tool improves the consistency in health condition detection systems and reduces the complexity of the diagnosis process [10]. A blockchain (BC)-based consistent sensor data analysis is implemented for health condition detection systems. BC analyzes the relevant data from a huge amount of wearable health data. The BC minimizes the energy consumption level in the detection process, enhancing the disease prediction process [11].

Safe distance estimation in crowded areas is a crucial task to perform in every healthcare system. The goal is to reduce the spread of contagious and infectious diseases between people in crowded public areas. Various methods and models are used for safe distance estimation in public places. A flexible modeling framework addresses the safest physical distance between people [12]. The modeling framework combines the health conditions produced by wearable sensor devices and traces the distance among unaffected people. The modeling framework provides different strategies using a surveillance monitoring system [13,14]. The physical distance scenarios are estimated based on people’s health conditions, improving the accuracy of the infectious disease detection process [15]. Real-time social distance measurement detection is also used in public places. The real-time distance among the individuals is identified using wearable sensor devices [16]. A deep learning (DL) algorithm is used here to identify the safety of people in public areas. DL increases the overall significance and reliability of the distance estimation process [17].

### Contributions

- The objective of this study is to provide a unique hardware/functional mechanism that may provide robust contagion safety recommendations despite variations in sensing accuracy and hardware failures. This architectural approach demonstrates an ability to dynamically adjust sensing intervals, thereby differentiating itself from previous designs. The utilization of a random forest classifier is employed to distinguish between consistent and inconsistent swings in health status, hence improving the precision of identifying individuals who are infected;
- This study proposes the implementation of a flexible sensing interval for hardware devices, which aims to maintain a consistent data observation process and provide appropriate suggestions. By mitigating the impact of time-series sensing fluctuations, this approach ensures the reliability and accuracy of the collected data;
- A thorough study of data and metrics was performed to verify the proposed system’s adherence to its design objectives.

## 2. Related Works

Ahmed et al. [18] proposed a social distance monitoring framework using deep learning (DL) architecture for COVID-19 infection control. The main aim of the framework is to identify the real-time physical distance among people in public places. The DL architecture trains the network to predict the safe distance using captured images. It improves the social-distancing range among people and reduces the infection level in the surroundings. The proposed framework increases the accuracy of person detection for infection control transmission.

Gevertz et al. [19] developed a new COVID-19 epidemiological model with an isolation compartment for social distancing. The developed model analyzes the flow of people and identifies the safe physical distance level. It also provides control strategies to protect people from contagious diseases. The isolation compartments are used for explicitly susceptible and asymptomatic populations. The developed epidemiological model reduces the overall negative effects of social distancing systems.

Abir et al. [20] introduced a pre-symptomatic detection framework based on DL and wireless data. A long short-term memory variational autoencoder (LSTM-VAE) is used in the framework to detect the anomalies. LSTM-VAE identifies the symptoms and conditions of the patients. Wireless data gathered from smart wearable devices reduces the complexity of the detection process. The introduced framework improves the feasibility and significance range of the detection process.

Youssef et al. [21] proposed a non-variant structuring (NVS)-approach-based supervised biosensor for infectious disease data analysis. The biosensor gathers patients’ health information from the healthcare system and wearable devices. The NVS approach classifies the health data based on infectious disease data. It is identified by grouping and categorizing the sensor data. The proposed approach increases the visualization and readability level of sensor data.

Ye et al. [22] designed an infectious disease monitoring framework with automated contact tracing. The actual goal of the framework is to detect infectious disease data from the given datasets. A tracing framework traces suspicious data via a monitoring process. It detects the psychological parameters and changes among patients. The designed framework improves the performance range of the diagnosis process in healthcare centers.

Kim et al. [23] introduced a successive epidemic prevention framework for intelligent public areas. Mobile robots and smart devices are used in the framework, which produces relevant information for monitoring systems. The introduced framework is mostly used in various applications to reduce problems in the prevention process. The introduced framework enhances the effectiveness level of physical distancing in public areas.

Hu et al. [24] focused on the significant issue of epidemic surveillance. They proposed an original technique that utilizes the Internet of Things (IoT) and an improved version of the gated recurrent unit (GRU) model. The primary aim is to enhance the precision and efficacy of monitoring the dissemination of diseases in real time. Internet of Things (IoT) technology facilitates the ongoing acquisition of data from many origins. At the same time, the sophisticated gated recurrent unit (GRU) model effectively captures intricate temporal patterns, augmenting predictive capacities. The system has several benefits, such as the ability to monitor in real time, improved accuracy in predicting outcomes, and the capacity to scale up for use in regional or global contexts. Nevertheless, certain apprehensions emerge concerning the preservation of data privacy, the necessary infrastructure prerequisites, and the computational intricacy associated with the model.

Davoodi et al. [25] addressed the constraints associated with conventional methodologies by proposing the integration of learning-based systems to transform hazard assessment and individual patient diagnoses. The main objective of this initiative is to address current disparities in healthcare practices through the integration of data-driven approaches, leading to enhanced precision in risk forecasting and significantly contributing to the progress of customized treatments. The contributions of this study are diverse, as they involve integrating machine learning techniques for hazard assessment, extending these methods to patient-specific diagnoses, and introducing the novel idea of multimodal data fusion. The improvements above hold the potential to facilitate proactive disease management, improve patient care, and foster a more thorough comprehension of disease dynamics. Nevertheless, the study also has some obstacles, including concerns about data privacy, the intricate nature of the model, and difficulties in interpreting the results.

Fu et al. [26] proposed a risk identification method based on complex network theory for major infectious disease epidemics (MIDE). Complex network theory is mainly used here to identify the risks from complex data. The MIDE selects the risks based on severity in the management process. It increases the accuracy of risk identification, which maximizes the feasibility of the systems. Experimental results show that the proposed MIDE method improves overall risk management.

Kim et al. [27] introduced a Bayesian spatiotemporal infectious disease model evaluation for public health surveillance. It evaluates the spatiotemporal features of infectious data and reduces the storage range of the systems. Both real and epidemic data are evaluated, improving the performance range of health surveillance systems. The introduced model predicts the exact features of infectious diseases via surveillance.

Arinik et al. [28] developed a new evaluation framework for epidemic surveillance systems. The main aim of the framework is to address the issues and problems in intelligent systems. It is mostly used in event-based surveillance (EBS) systems, which have various problems with respect to their management. The framework detects both spatial and temporal sources via the analysis process. The developed framework maximizes the accuracy of the parameter evaluation process.

Liu et al. [29] proposed a spatial tessellation algorithm for epidemic decision support. A gradient learning algorithm is used here to locate the tessellation, reducing the latency in further processes. An estimation process evaluates the necessary features and parameters for decision support systems. The proposed algorithm improves the effectiveness and feasibility range of the decision-making process.

Rusin et al. [30] introduced a new COVID-19 forecast epidemic detection method for small districts. The short-term forecast is evaluated to gather optimal information for epidemic evolution. It minimizes the complexity of contagious disease spread levels in the districts. The actual differences among the data are identified for the decision-making process. Compared with other methods, the proposed method increases the accuracy of epidemic detection. According to the researcher, contagious disease infections are identified using machine learning techniques such as neural networks, support vector machines, and neural networks. These intelligent techniques effectively work on human crowd information. However, the methods require a large volume of data to train and improve the classifier performance. Also, there are complexities while examining the spread level in districts. These research issues are overcome by creating safety recommendation systems that successfully prevent contagious disease infections.

## 3. Data Preface

**Figure 1:**
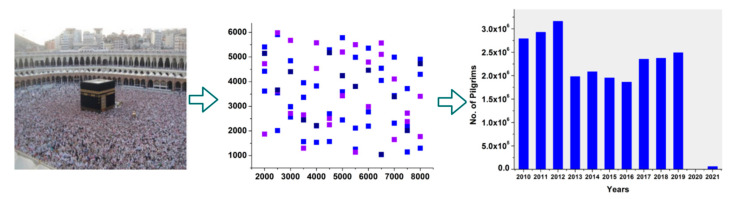
Illustration of density distribution and drop analysis.

The proposed method was validated using the UCF_QNRF [31] dataset. The dataset provides a Hajj crowd count from different archives. The crowd count was based on 1535 images from web sources, recorded inputs, etc. The cumulative images identified 1,251,642 visitors in a specific year. However, a change in density occurred after the COVID-19 pandemic. Figure 1 illustrates the density distribution and its drop over the years.

The average crowd density distribution is 1.12$\;\times \;10^{-4}$ [31], represented by an average of 815 headcounts. The drop-in pilgrims in Haji can be observed in the third image, as given in (https://www.statista.com/statistics/617696/saudi-arabia-total-hajj-pilgrims/, accessed on 20 August 2023). This is due to the pandemic and the safety measures observed in 2019 and 2021. Therefore, regardless of the density, public safety was prioritized and implemented in 2021 compared to 2019. Our proposed model augments the priority for public safety using hardware and software combinations.

### 3.1. Consistency-Focused Recommendation System (CRS) for Personal Healthcare (PH)

**Figure 2:**
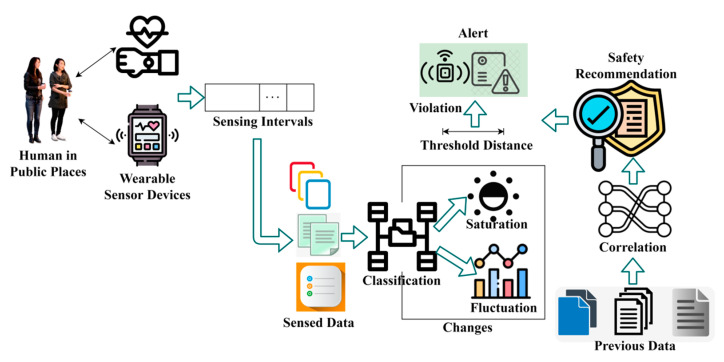
Structure of consistency-focused recommendation system.

The proposed recommendation system is designed to maintain a safe distance between infected and non-infected people and reduce contagious disease spread in human crowds. The wearable sensor devices are placed over the human body for observing data. In this proposed system, the data are sensed or observed by people using wearable sensors, and communication technologies and recommendation modules are used for contagious disease spread identification. Personal healthcare systems are designed to avoid sensing fluctuations and hardware discharges by adjusting sensing intervals. The consistency of the human health status varies based on their movements or other activities identified through sensors. The sensor is a mixture of hardware and software components used to observe and analyze data accumulated from humans in public places. The processes involved in the proposed method are illustrated in Figure 2.

The proposed method is the design of a hardware/functional process for providing safety recommendations for people about contagious diseases regardless of sensing fluctuations and hardware discharges. The wearable sensor devices are equipped with sensing units to observe data, such as temperature, blood pressure, heartbeat rate, virus spread, etc., which are continuously monitored to prevent the spread of contagious diseases. This hardware architecture differs from previous data analysis by adjusting sensing intervals more flexibly to identify and separate consistent and inconsistent fluctuations in human health statuses for the precise recognition of infected people using a random forest classifier. In a flexible sensing interval, wearable sensor devices are used to achieve consistent data observation and optimal safety recommendations, regardless of time-series sensing fluctuations. The observed data are exploited to control the spread of contagious diseases. The proposed CRS, used for precise sensed data assessment and threshold distance, was devised using safety recommendations for people. Separate safety recommendations for infection-affected and non-affected people are used to maintain threshold distance. The hardware sensing intervals are calibrated using conventional specifications in which abrupt variations can be observed in humans. 

A consistent healthcare safety recommendation system for controlling infection spread between people, this system helps to prevent contagious disease infection in human crowds. Assume that {P=1,2,3,…n} represent the total population. The infection-affected people are denoted as ${\;P}^{inf}$ and non-infected people ${\;P}^{non_{inf}}$ through wearable sensor devices. The function of the devices is controlled and managed by wireless signals and recommendation modules. The CRS method for PH performs between sensing units and people. The violation in threshold distance is identified using wearable sensor devices, where saturation and fluctuation changes are precisely identified and classified. The classification of saturation and fluctuations using a random forest classifier for controlling disease spread is obtained using the inputs collected from the wearable sensor devices $\left(\varepsilon \right)$. The sensors observe people’s temperature/blood pressure or heartbeat rate, etc., to recognize contagious disease infection spread. The data observed from different periodic sensing intervals are analyzed through $\left(\varepsilon \right),$ and appropriate action is taken to control infection spread. Hence, the CRS is modeled into two segments: the sensed data assessment and threshold distance for the moving user.

### 3.2. Sensed Data Assessment

The deployed wearable sensor devices are responsible for continuously sensing and monitoring people’s health status in public places. The observed input data can be related to temperature, blood pressure, and heart rate. In a sensing instance, the sensed input data processing $\left({SI}_{d}\right)$ is computed as

(1) $${SI}_{d}=\sum_{\varepsilon_{id}=1}P^{{non_{inf}}_{i}}-P^{{inf}_{i}}$$

such that

(2) $${∆}^{sf}=\frac{1}{\sqrt{2\pi }}\left[\frac{\sum_{\varepsilon_{id}=1}\left(\frac{P^{inf}}{P^{non_{inf}}}\right)}{2\left({SI}_{d}-{PSI}_{d}\right)}\right]$$

and

(3) $${Hw}^{Disch}=\left(\varepsilon_{id}-\frac{{\left(C^{int}\right)}_{min}}{{\left(In_{C}^{int}\right)}_{\max}}\right)$$

**Figure 3:**
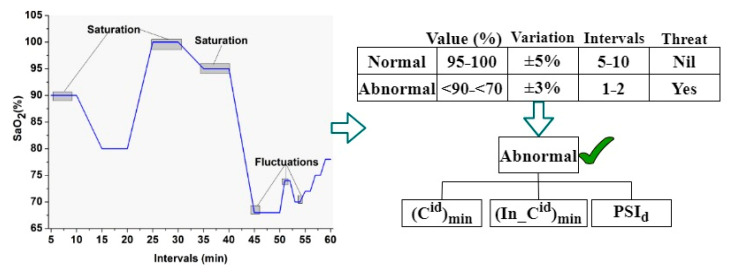
Graphical analysis of sensing fluctuation from real-time data.

Based on the Equations (1)–(3), the variable $\varepsilon_{id}$ denotes the sensor identity number and n∈ε_id, $P^{{non_{inf}}_{i}}$d $P^{{inf}_{i}}$e the non-infected and infected people identified by the sensed data at different intervals. The variables ${∆}^{sf}$, ${Hw}^{Disch}$ and ${PSI}_{d}$ used to represent the sensing fluctuations, hardware discharges, and the person’s previous, healthy record data. Where the variables ${\left(C^{int}\right)}_{min}$ and ${\left(In_{C}^{int}\right)}_{max}$ used to represent minimum and maximum consistent and inconsistent fluctuations in health status for precise contagious disease infection detection. An illustration of the sensing fluctuations is given in Figure 3. For an efficient illustration of the fluctuation, the data from [32] are exploited in this representation. This data provides oxygen, false rates, and temperatures of humans (in public) measured using wearable sensors. The fluctuations are provided at different intervals of oxygen levels. The data are assumed to be augmented with the Hajj data, wherein the sensing process is the same.

The fluctuations and saturations are observed over the sensing intervals based on the normal and abnormal values. If the fluctuations regain the normal values, fewer variations than the $SaO_{2}$ marks a human as normal. This is similar to the oxygen, pulse rate, and temperature data used when detecting pandemic infectious diseases like COVID-19. If any abnormal cases of wearable sensor observation are sensed, then ${\left(C^{int}\right)}_{\min\;}n$ and $\left(In-C_{min}^{int}\right)$ are identified based on conventional clinical data. In this process, $PSI_{d}$ is useful in detecting a person’s health condition in previous and current sensing intervals (Refer to Figure 3 Representation). Sensor fluctuations are computed as the number of mismatched health records observed at different intervals. In this case, some errors can occur in ${SI}_{d}$ due to consistent and inconsistent sensing interval issues. Therefore, these issues affect the sensed data at any intervals for which the normalization is estimated as:

(4) $$Normz\left({SI}_{d}\right)=\frac{X^{2}}{{\left(\frac{{\left(C^{int}\right)}_{min}}{{\left(In_{C}^{int}\right)}_{\max-Y}}\right)}^{2}}$$

and

(5) $$Y=\frac{1}{\varepsilon_{id}}\sqrt{\frac{1}{{∆}^{sf}-1}\sum_{i=1}^{\varepsilon_{id}}{\left(\frac{{SI}_{d}-{PSI}_{d}}{C^{int}-In_{C}^{int}}\right)}^{2}}$$

where the variables X and Y used to denote saturation and fluctuations at the time of the information sensing intervals, the sensed data are classified as saturation and fluctuations based on the changes in sensing intervals. Equations (4) and (5) compute the normalization of sensed data in different instances following the maximum or minimum saturation and fluctuation intervals based on the sensor values. Here, X is a saturation value whereas Y is the fluctuation value, for which the accurate computation of ${SI}_{d}$ is required for precise detection. Based on ${SI}_{d}$ and $Normz\left({SI}_{d}\right)$, the continuous changes are estimated as:

(6) $$\exists \left[{SI}_{d},Normz\left({SI}_{d}\right)\right]=\left\{\begin{array}{c} {\left[\frac{Normz\left({SI}_{d}\right)}{{SI}_{d}}\right]}_{1}^{2}\\ +\\ {\left[\frac{Normz\left({SI}_{d}\right)}{{SI}_{d}}\right]}_{2}^{2}\\ +\\ \vdots \\ +\\ {\left[\left(1-\frac{{PSI}_{d}}{{SI}_{d}}\right)X\right]}_{\varepsilon_{id}}^{2}\; \end{array},\right.n\in \varepsilon_{id}$$

**Figure 4:**
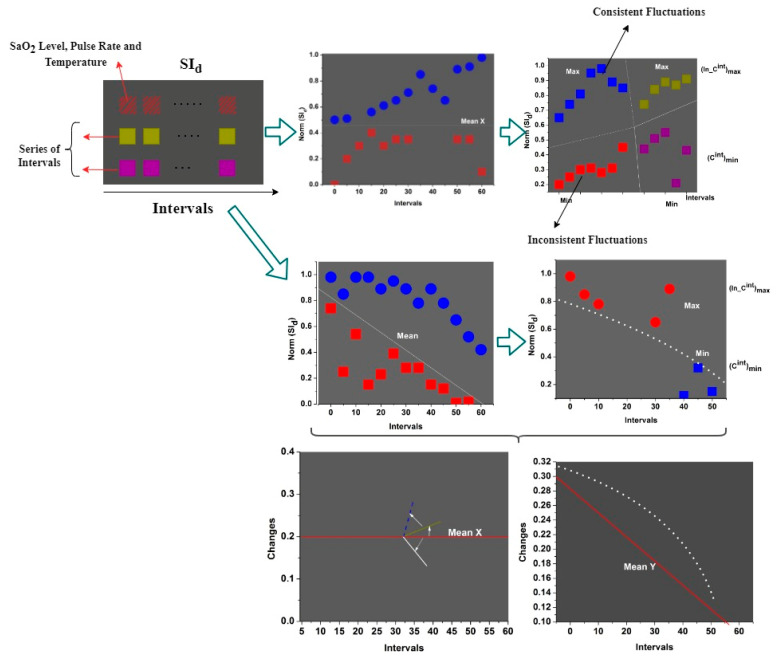
Classification analysis for abrupt change detection.

Equation (6) computes the consistency fluctuations for a sequence until wearable sensor devices are active in processing sensed data from humans in public places. The current data handling and processing for precise contagious disease detection is used until the device requires sensing. The above sequence of changes is identified using a random forest classifier. In this scenario, the sensed data will be classified as saturation, and fluctuations observed from the neighbors within the threshold region are identified to control the contagious disease spread. The safety recommendations must be distributed at appropriate and accurate sensing intervals to improve consistency and saturation. The infected people are isolated and maintain a threshold distance between people. Contrarily, consistency is checked for a sequential row of intervals for the fluctuating sensed data. This is performed to alert people when the threshold distance for a contagious disease is violated. In addition, the threshold distance is instantaneous to control infection spread. Therefore, the random forest classifier is used for abrupt change identification. The classification for abrupt change detection is presented in Figure 4.

The above classification was used for $(In\;\_C^{int}{)}_{max}$ and ${\left(C^{int}\right)}_{min}$ across various sensing intervals. This classification was performed for the sensed $SaO_{2}$ level, pulse rate, and temperature to determine the abrupt change behind max and beneath min for the mean *X* and *Y*. This mean value was obtained from the Norm $\left(SI_{d}\right)$ by identifying $n\in \rho^{non\;\_inaf_{i}}$ or $P^{inf_{i}}$. However, these two points were used as input for the classification learning as M and N. In the initial case, M represents the sensed $n\in \varepsilon_{id}$ and N=0; the saturation and fluctuation classification, as in Figure 3, was performed (Figure 4 illustration). The output of the classifier was used to identify and segregate the consistent and inconsistent fluctuations of the identified health status through the ${SI}_{d}$ assessment and ${PSI}_{d}$ correlation. The correlation of the current person’s health data and the previous health record was used to achieve $\left(1-\frac{{PSI}_{d}}{{SI}_{d}}\right)X$ in the first assessment as the precise output for providing safety recommendations to people. For this purpose, two consecutive samples of ${SI}_{d}$ at different sensing intervals were assessed. Let M and N are serving as the input for the random forest classifier. To differentiate the above from the previous healthy data sensed, the sensing intervals were modeled as per Equation (7):

(7) $$\left.\begin{array}{c} M={SI}_{d}\\ N=0 \end{array}\right\},\;as\;the\;first\;interval\;observed$$

and

(8) $$\left.\begin{array}{c} M=Normz\left({SI}_{d}\right)\\ N=\frac{Y}{X} \end{array}\right\},\;for\;the\;series\;of\;intervals$$

such that

(9) $$\left.\begin{array}{c} M+N={SI}_{d},\;as\;the\;first\;input\;sample\\ and,\\ \;M+N=Normz\left({SI}_{d}\right)+\frac{Y}{X},\;is\;the\;input\;for\;series\;of\;intervals \end{array}\right\}$$

**Figure 5:**
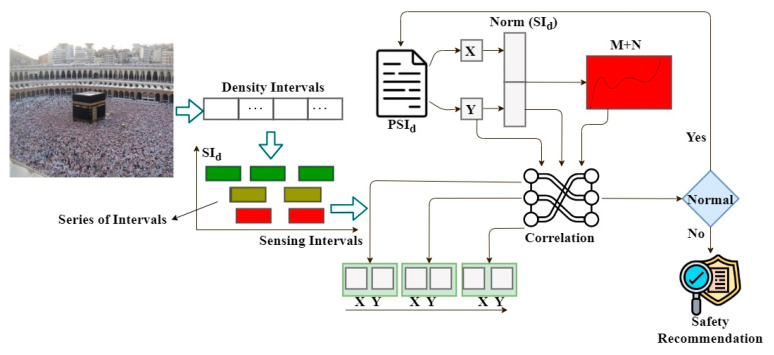
Correlation process of post classification.

The random classifier computation was initiated from the series of intervals with the first training set as ${SI}_{d}$. The ${SI}_{d}$ represented high-consistency sensed intervals; if any fluctuations (later) were observed, a threshold distance is provided to the hardware-identified location. Hence, in the random classifier, the series of intervals $M+N=Normz\left({SI}_{d}\right)+\frac{Y}{X}$ accounted for addressing the saturation and fluctuation based on sensor values. The classifier consisted of two stages: classification and correlation, followed by the output. The function of fluctuations treated by the random classifier was defined as per Equation (8)

(10) $$\mu \left\{\exists \left[{SI}_{d},Normz\left({SI}_{d}\right)\right]\right\}=-\left(M\exists^{O}+N{SI}_{d}+\exists^{O}{SI}_{d}\sigma^{O}\right)$$

such that

(11) $$\left.\begin{array}{c} M\left(\exists^{O}|{SI}_{d}\right)={SI}_{d}+\sigma^{O}\exists^{O}\\ N\left({SI}_{d}|∆\right)=M-\sigma^{O}\exists^{O} \end{array}\right\}$$

where $\exists^{O}$ is the classification output, and $\sigma^{O}$ is the correlation output observed from the sensing intervals by mapping ${SI}_{d}$, M,N with $\exists^{O}$. In the above equation, $M\left(\exists^{O}|{SI}_{d}\right)$ and $N\left({SI}_{d}|∆\right)$ were the properties used for satisfying the condition $\mu \left\{\exists \left[{SI}_{d},Normz\left({SI}_{d}\right)\right]\right\}$. As defined in the above Equation (8), the function of sensing fluctuations either satisfies $M\left(\exists^{O}|{SI}_{d}\right)$ or $N\left({SI}_{d}|∆\right)$. The correlation process post-classification is illustrated in Figure 5.

The correlation pursues three validations against the $\left(X,Y\right)$ combinations. The first is the $M=SI_{d}$ and N=0 case followed by Norm $\left(SI_{d}\right)$ for fluctuation observed inputs. In contrast to these cases the $\left(M+N\right)$ is the classified input for correlation. All three cases are used for identifying $p^{inf_{i}}$ using $n\in \varepsilon_{id}$ under normal and abnormal classifications. If the normal case is observed, then $PSI_{d}$ is updated, or else the safety recommendation is provided. This safety recommendation follows the international guidelines for infectious disease spread prevention (Figure 5 Reference). The consistent and inconsistent fluctuations of the above representation generate the linear output of M±N to satisfy the abovementioned properties. The fluctuation properties of $M\left(\exists^{O}|{SI}_{d}\right)$ and $N\left({SI}_{d}|∆\right),$ and the inconsistent fluctuations in health status are identified using X and $Normz\left({SI}_{d}\right)$ to jointly produce the output of the correlation at the most precise point of contagious disease detection. In this manner, the random classifier follows M, N and $\exists \left[{SI}_{d},Normz\left({SI}_{d}\right)\right]$ by the correlation to provide safety recommendations for moving people through ${PSI}_{d}$ and X analysis.

As represented in the first sensing interval and series of sensing intervals, the correlation process is then performed using the classification output. In the first instance, the fluctuation properties are defined for identifying accurate changes in human health status, and its consistency measure is used for providing safety recommendations. Hence, the sensor value of the first user is retained without saturation and fluctuations. Instead, in the sequential instances, it varies wherein the differentiation in fluctuations $M(\exists^{O}|X)$ and $N(\exists^{O}|X)$ impacts the consistency factor. In this analysis, the occurrence of the above properties identified in any intervals either satisfies $M(\exists^{O}|X)$ or $N(\exists^{O}|X)$. This is because the saturation and fluctuations of the inputs are classified for precise contagious disease detection such that the probability of sensing fluctuation is 0.5 for the intervals. Based on this analysis, the $\sigma^{O}$ conditions in $\sigma^{O}>\frac{Y}{X}$ or $\sigma^{O}\leq \frac{Y}{X}$ are assessed for providing safety recommendations and for maintaining a safe distance between people to avoid contagious disease spread. Using Equation (12), the correlation output $\sigma^{O}$ and its consistency is given as:

(12) $$\sigma^{O}=1-\frac{\rho_{C^{int}}}{\rho_{In\_C^{int}}}$$

In Equation (12), the variables $\rho_{C^{int}}$ and $\rho_{In\_C^{int}}$ represent the probability of consistent and inconsistent fluctuation health status of the identified and separated people to avoid disease spread based on sensor values. It is to be noted that not all sensing intervals can be associated with both M and N. Then, the classification output based on the conditions $\sigma^{O}>\frac{Y}{X}$ and $\sigma^{O}\leq \frac{Y}{X}$ is computed as

(13) $$\left.\begin{array}{c} \exists^{O^{1}}=Normz{\left({SI}_{d}\right)}_{1}\\ \exists^{O^{2}}=Normz{\left({SI}_{d}\right)}_{2}-{\left(\frac{Y}{X}\right)}_{1}-{\left(\frac{\rho_{C^{int}}}{\rho_{In\_C^{int}}}\right)}_{1}\\ \begin{array}{c} \exists^{O^{3}}=Normz{\left({SI}_{d}\right)}_{3}-{\left(\frac{Y}{X}\right)}_{2}-{\left(\frac{\rho_{C^{int}}}{\rho_{In\_C^{int}}}\right)}_{2}\\ \begin{array}{c} \vdots \\ \exists^{O^{i}}=Normz{\left({SI}_{d}\right)}_{i}-{\left(\frac{Y}{X}\right)}_{i-1}-{\left(\frac{\rho_{C^{int}}}{\rho_{In\_C^{int}}}\right)}_{i-1} \end{array} \end{array} \end{array}\right\},\;\sigma^{O}>\frac{Y}{X}$$

(14) $$\left.\begin{array}{c} \exists^{O^{1}}={SI}_{d_{1}}-N{\left({SI}_{d}|X\right)}_{1}\\ \exists^{O^{2}}={SI}_{d_{2}}-N{\left({SI}_{d}|X\right)}_{2}-{\left(\frac{P^{inf}}{P^{non\_inf}}\right)}_{1}\\ \begin{array}{c} \exists^{O^{3}}={SI}_{d_{3}}-N{\left({SI}_{d}|X\right)}_{3}-{\left(\frac{P^{inf}}{P^{non\_inf}}\right)}_{2}\\ \vdots \\ \exists^{O^{i}}={SI}_{d_{i}}-N{\left({SI}_{d}|X\right)}_{i}-{\left(\frac{P^{inf}}{P^{non\_inf}}\right)}_{i-1} \end{array} \end{array}\right\},\;\sigma^{O}\leq \frac{Y}{X}$$

**Figure 6:**
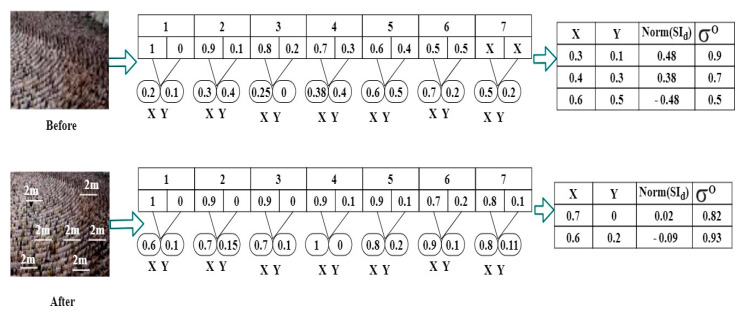
Analysis of correlation and normalization outcomes.

The above correlation output follows for the i series of intervals, where normalization and input sensed data are the augmenting factors for the differentiation of the output of the random classifier. Then, the sensing fluctuation is verified for changes in the above conditions of $\sigma^{O}>\frac{Y}{X}$ and $\sigma^{O}\leq \frac{Y}{X}$ using the assessment below. The correlation and normalization outcomes are illustrated in Figure 6 based on the classification for the two conditions above.

The above representation was obtained from the dataset for which two cases were considered. The conventional crowd is represented in the “before” images and the safety precaution-based implemented recommendations are visualized in the “after” images. The safe distance is implemented in the “after” image. Both cases are validated for X,Y and $\sigma^{o}$, for which the $N\left(SI_{d}\right)$ is performed. If the density is high, the X variation is high compared to the “after” density. As the X variations are high, the Norm $\left(SI_{d}\right)$ is high for reducing fluctuations. In the contrary case of “after”, the safety recommendations are high, and therefore X is high compared to Y. This requires less $Norm\;\left(SI_{d}\right)$ and a high $\sigma^{o}$ with $PSI_{d}$ for better recommendations (Illustrations in Figure 6).

### 3.3. Hardware Discharges

In the final hardware discharge analysis, the sensing unit exploits the sensor data analysis to determine the function and operation of the wearable sensor devices. Let the initial hardware discharge ω=0 such that, if ω=1, then the sensors are operated. This relies on $\sigma^{O}$ and $\mu \left\{\exists \left[{SI}_{d},Normz\left({SI}_{d}\right)\right]\right\}$ such that the probability of the hardware discharge $\left(\rho_{hwdc}\right)$ is given as:

(15) $$\rho_{hwdc}=\frac{{\left[count\;\left(\frac{P^{inf}}{P^{non\_inf}}\right)\right]}^{\varepsilon_{id}}\times {\left(C^{int}\right)}_{min}-{\left(In\_C^{int}\right)}_{max}{)}^{i-1}}{\sum_{s\in S}{\left[count\left(\frac{P^{inf}}{P^{non\_inf}}\right)\right]}^{\varepsilon_{id}}\times (1-\sigma^{O}{)}^{i-1}}$$

**Figure 7:**
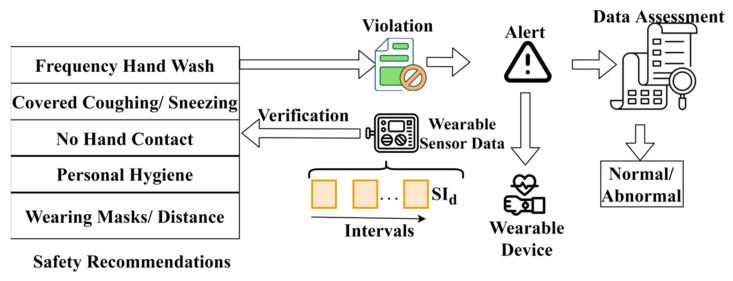
Process of violation detection and alert.

From Equation (15), the probability of hardware discharge is computed to ensure that the devices work. If any problem is identified in the hardware, then immediate changes occur in the sensor to avoid disease spread. The segregation of consistent and inconsistent fluctuations in health records helps to identify disease spread between people. The Saudi embassy provided a safety recommendation for Hajj pilgrims after the COVID-19 pandemic. The correlation process relies on the fluctuations and variations observed. This is required for verifying violations in the crowd and providing alerts. This alerting process is identified for safety recommendations from the government guidelines. This process is illustrated in Figure 7.

The safety recommendations are validated along $SI_{d}$ (wearable sensor data) for identifying violations. The alert is provided if any violation is observed, and the data obtained during the interval is used for the assessment. This assessment provides prior information on normal/abnormal statuses. Therefore, the dual information is cumulatively used for validating the personal health status and preventive measures (Figure 7).

## 4. Results and Discussion

This section continues the above discussion using specific metrics and methods. The metrics are related to the findings, novel discussions, and briefings presented in the proposed method’s section. Therefore, the related metrics, including the false rate, fluctuations, data analysis rate, recommendation ratio, and consistency checks are comparatively analyzed. The average observation/person and observation intervals are the variants for data validation. The methods GA-GRU (genetic algorithm-based gated recurrent unit) [25], GVT (greedy–Voronoi tessellation) [29], and PCovNet (pre-symptomatic COVID-19 detection framework) [21] were used alongside the proposed CRS-PH method in the comparative analysis. 

### 4.1. False Rate

The false rate is the misinterpretation of $n\in P^{non\;\_inf_{i}}$ as $n\in p^{inf_{i}}$ or vice versa. This interpretation is handled using $\left(M,N\right)$ such that $\sigma^{o}>\frac{y}{X}$ or $\sigma^{o}\leq \frac{Y}{X}$ is satisfied. For the false rate mitigation, the following steps are used:

$$Step\;1:compute\;\exists^{o^{i}}=Norm\;{\left(SI_{d}\right)}_{i}-{\left(\frac{Y}{X}\right)}_{i-1}-{\left(\frac{\rho_{C^{int}}}{PIn\;\_C^{int\;}}\right)}_{i-1}$$

$$Step\;2\;if\;\sigma^{o}>\frac{Y}{X}then$$

$$Step\;3:M\left(\exists^{o}|SI_{d}\right)=SI_{d}+\sigma^{o}\exists^{o}$$

This reduces the false rate without $N\left(SI_{d}|∆\right)$ by mapping M,N separately.

**Figure 8:**
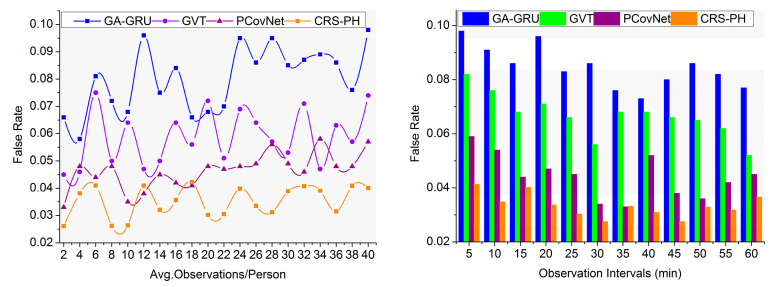
Comparative analysis of false rate.

This consistency-focused recommendation system achieves fewer false rates by analyzing the changes for differentiating the saturation and fluctuations observed from the neighbor within the threshold distance, as represented in Figure 8. The sensing fluctuations and hardware discharges are continuously monitored and identified for computing the consistency for proving appropriate safety recommendation to reduce the false rate. This analysis is performed when classifying saturation and fluctuations at different sensing intervals for augmenting recommendation system efficiency. The new safety recommendations are generated based on human health status observed from the devices evaluated for precise contagious disease detection, preventing computation complexity. Abrupt changes are observed in the calibrated hardware sensing intervals as per the specifications. This process is performed to ensure a maximum recommendation ratio for analyzing the sensed data, which is used for providing an alert signal to the user to avoid contagious disease spread. Thus, the proposed system verifies consistency and inconsistent fluctuation intervals to maintain a safe distance between people, for which the false rate is reduced. The proposed method reduces the false rate by 10.87% and 8.56% for the above variants.

### 4.2. Fluctuations

**Figure 9:**
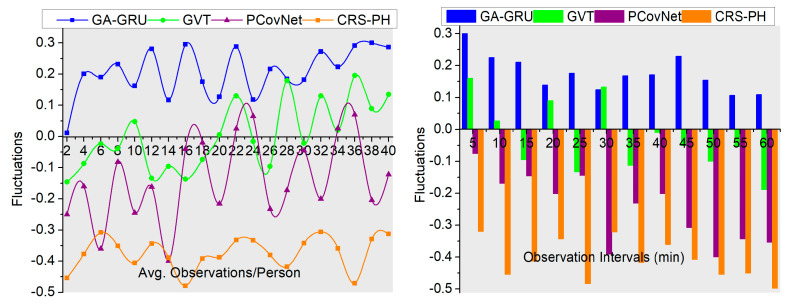
Comparative analysis of fluctuations.

This proposed system reduces assessment time and fluctuation occurrence in sensing intervals based on observing human movement and other activities. The personal recommendations are analyzed using wearable sensor signals, and their consistency is sensed to identify changes; this analysis is pursued to reduce identification errors, computation complexity, and fluctuations by applying a random forest classifier. The hardware sensing intervals are sequentially analyzed in both instances to prevent fluctuations. The recommendation system for controlling contagious disease spread in a human crowd is performed to identify and separate consistent and inconsistent fluctuations. Based on the classification output, the identification error is easily identified, and the correlation of the current user’s health record with the previous health record provides accurate safety recommendations for preventing additional analysis time and inconsistency. The personal healthcare systems are designed to avoid sensing fluctuations and hardware discharges by adjusting the sensing intervals. The consistency of the human health status varies based on the user’s movements or other activities identified through sensors for maximizing the recommendation ratio. In this proposed system, consistency verification is performed to achieve fewer fluctuations, as illustrated in Figure 9. For the variants considered above, the proposed method reduces fluctuations by 6.88% and 7.07%.

### 4.3. Data Analysis Rate

**Figure 10:**
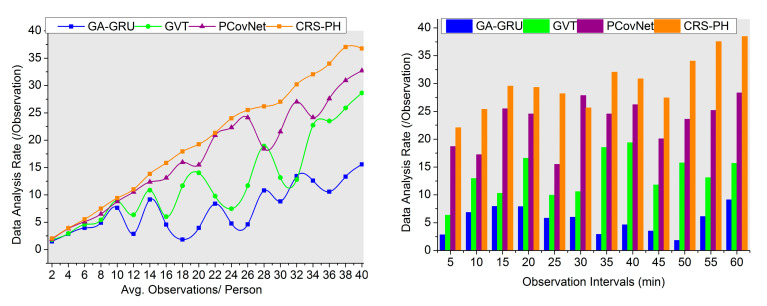
Comparative analysis of data analysis rate.

This article achieves a high data analysis rate for different sensing intervals to maximize the safety recommendations to reduce contagious disease spread (refer to Figure 10). Errors and assessment times are mitigated through the classification process for precise infection spread identification. The high data analysis rate is achieved due to the series of intervals for the fluctuating sensed data. This proposed system uses the design of a hardware process for providing safety recommendations for people against contagious diseases regardless of sensing fluctuations and hardware discharges in different instances. The sensed data are processed to classify saturation and fluctuations in sensing intervals to address the false rate. The series of intervals are analyzed for sensing fluctuations and hardware discharges for improving safety recommendations through classification to reduce the computational complexity in both instances. Similarly, by augmenting a flexible sensing interval, the wearable sensor devices used for achieving consistent data observation and optimal safety recommendations regardless of time-series sensing fluctuations, achieve a high data analysis rate. The CRS-PH augments the data analysis rate by 10.09% and 8.9%, respectively.

### 4.4. Recommendation Ratio

The recommendations are provided under the following considerations:

$$\;Step\;1:Compute\;{\left(C^{int}\right)}_{min}\;\;and{\;(In\;\_C)}_{max}\;\mathrm{as}\;\mathrm{per}\;\mathrm{Equation}\;(3)$$

$$\;step\;2:if\;\left(X>Y\right)then$$

Step 3:N=0 else

$$Step\;4:M+N=SI_{d}$$

$$step\;5:If\;P_{hwdc}=1\;\forall M+N=SI_{d}\;\mathrm{then}$$

$$step\;6:Provide\;recommendations\;of\;PSI_{d}\;with\;SI_{d}$$

**Figure 11:**
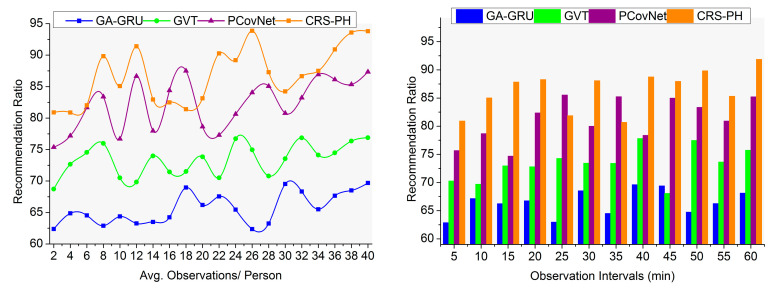
Comparative analysis of recommendation ratio.

This proposed system for primary healthcare achieves a high recommendation ratio based on detecting the changes in saturation and fluctuations observed from a neighbor within the threshold distance for reducing disease spread (refer to Figure 11). The consistent and inconsistent sensing intervals fluctuate based on changes observed in the instance are analyzed for improving saturated data and its consistency. This sensed data from humans is analyzed to control contagious disease spread in public places. The proposed CRS used for consistent data observation and precise sensed data assessments was devised to provide safety recommendations for the people to overcome this situation. Separate safety recommendations are provided for infection-affected and non-affected people to maintain a safe distance. Therefore, consistent data observation is performed to improve the recommendation system’s efficiency. Violations in threshold distances are identified using wearable sensor devices, where saturation and fluctuation changes are accurately identified. In this article, the defined ε aided in identifying an accurate status for a person to achieve a high recommendation ratio. The recommendation ratio was improved by 15.83% and 15.5% for the above X-Axis variants.

### 4.5. Consistency Check

In the data consistency check ${\left(C^{int}\right)}_{min}$ and In_C^int)max is verified ∀i∈n. This validation comes across $P^{inf_{i}}$ and $p^{non\_inf_{i}}$ such that $M\left(\exists^{o}|SI_{d}\right)$ and $N\left(SI_{d}|∆\right)$ are identical. Therefore:

$$Step\;1:Compute\sigma^{o}\;as\;in\;Equation\;(12)$$

$$Step\;2:if\;\sigma^{o}>\frac{Y}{X}\;then$$

Step 3:Perform μ for consistency;else

$$step\;4:M=SI_{d},N=0$$

$$step\;5:Compute\;{∆}^{sf}$$

$$step\;6:Goto\;step\;2\;\mathrm{and}\;\mathrm{continue}\;\mathrm{until}\;\sigma^{o}\leq \frac{Y}{X}is\;achieved$$

**Figure 12:**
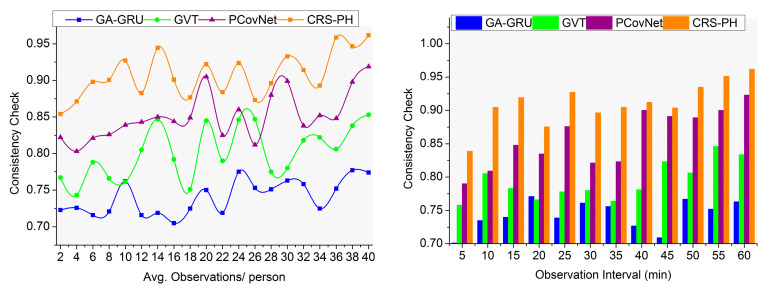
Comparative analysis of consistency check.

This manuscript performs the consistency verification process accurately compared to the other factors for preventing contagious disease spread (refer to Figure 12). In this proposed system, the classification of saturation and fluctuations is performed for detecting computational complexities and false rates for achieving highly consistent data observations and optimal safety recommendations. Based on the condition, the sensing fluctuation variations are identified as the number of mismatching health records observed. In this case, some false rates can occur in $SI_{d}$ due to changes in consistency. The above sequence of changes is identified using a random forest classifier to prevent false rates. In this scenario, the sensed data will be classified into saturation, and fluctuations observed from the neighbors within the threshold region are identified to control contagious disease spread. The safety recommendations must be distributed for changes identified by the hardware for improving consistency through recommendations. In this proposed system, consistent data observation depends on sensing intervals, for which a high consistency check was performed in this article. The consistency in checking was improved by 11.22% and 12.19%, respectively.

## 5. Discussions

The practical importance of the proposed “Consistent Healthcare Safety Recommendation System for Preventing Contagious Disease Infections in Human Crowds” (CRS-PH) is highlighted through a comparison study with existing methods such as GA-GRU, GVT, and PCovNet. The findings obtained from this analysis demonstrate the remarkable effectiveness of the CRS-PH system. The CRS-PH approach exhibits a notable decrease in erroneous rates, exceeding the performance of GA-GRU and GVT by 10.87% and 8.56%, respectively. Additionally, the system significantly reduces fluctuations by 6.88% and 7.07% for the examined variations, underscoring its resilience in delivering reliable predictions. The CRS-PH technique demonstrates a significant enhancement of 15.83% and 15.5% for the specified X-Axis variants, significantly improving the recommendation ratio. This ratio is a crucial statistic in the evaluation of public health interventions.

Additionally, the suggested system has exceptional performance in enhancing data analysis rates by 10.09% and 8.9%, thus highlighting its efficacy in efficiently processing and understanding data to facilitate prompt decision-making. The CRS-PH approach demonstrates notable advancements in consistency in checking, with improvements of 11.22% and 12.19% observed, respectively. The findings provide strong evidence for the higher effectiveness of the CRS-PH system, suggesting its capacity to strengthen strategies for preventing infectious diseases by reducing false positives, improving suggestion accuracy, and ensuring greater consistency in data analysis.

## 6. Conclusions

In this article, a consistency-focused recommendation system for personal healthcare was introduced. The research study improves the accuracy and flexibility of contagious disease detection by incorporating infection alerting systems and healthcare systems using adaptable calibrations. Regardless of the sensed output fluctuations that impact the distance factor, this proposal identified consistent infection data rather than periodic alerts. The random forest classification was used to identify saturations and fluctuations post the sensed data. By correlating the previous health data, the current health condition of a person in a public crowd could be assessed. The abnormal wearable sensor observations are monitored for their variations, from which an abnormality level is determined. Based on this level, further diagnosis or a personal health recommendation can be provided. In addition, the patient’s condition can be shared with neighbors, violating the pandemic-prevention rules through alerts. This method proposes an alert based on the analyzed data for preventing a healthy person’s physical contact with an abnormal person. This process is coordinated with the hardware and data computing processes pursuing the safety recommendation for preventing infectious disease spread. However, the system faces difficulties due to ethical considerations, which reduce the entire system’s reliability. In the future, long-term validations of user-centric design require the public health authority’s collaboration to effectively deploy the system.

## Data Availability

Data are contained within the article.

## References

[1] Mehmood I., Li H., Qarout Y., Umer W., Anwer S., Wu H., Hussain M., Fordjour Antwi-Afari M. (2023). Deep Learning-Based Construction Equipment Operators’ Mental Fatigue Classification Using Wearable EEG Sensor Data. Adv. Eng. Inform..

[2] Geng Y., Cao R., Innocent M.T., Hu Z., Zhu L., Wang L., Xiang H., Zhu M. (2022). A High-Sensitive Wearable Sensor Based on Conductive Polymer Composites for Body Temperature Monitoring. Compos. Part A Appl. Sci. Manuf..

[3] de Fazio R., Giannoccaro N.I., Carrasco M., Velazquez R., Visconti P. (2021). Wearable Devices and IoT Applications for Symptom Detection, Infection Tracking, and Diffusion Containment of the COVID-19 Pandemic: A Survey. Front. Inf. Technol. Electron. Eng..

[4] Kouis P., Michanikou A., Anagnostopoulou P., Galanakis E., Michaelidou E., Dimitriou H., Matthaiou A.M., Kinni P., Achilleos S., Zacharatos H. (2021). Use of Wearable Sensors to Assess Compliance of Asthmatic Children in Response to Lockdown Measures for the COVID-19 Epidemic. Sci. Rep..

[5] Lim W.J., Abdul Ghani N.M. (2022). COVID-19 Mandatory Self-Quarantine Wearable Device for Authority Monitoring with Edge AI Reporting & Flagging System. Health Technol..

[6] Burton S., Landers T., Wilson M., Ortiz-Gumina C., Persaud A., McNeill Ransom M., Fox L., Murphy S.A. (2023). Public Health Infection Prevention: An Analysis of Existing Training during the COVID-19 Pandemic. Public Health.

[7] Xiong L., Hu P., Wang H. (2021). Establishment of Epidemic Early Warning Index System and Optimization of Infectious Disease Model: Analysis on Monitoring Data of Public Health Emergencies. Int. J. Disaster Risk Reduct..

[8] Majeed A., Hwang S.O. (2021). A Comprehensive Analysis of Privacy Protection Techniques Developed for COVID-19 Pandemic. IEEE Access.

[9] Wang R., Ji C., Jiang Z., Wu Y., Yin L., Li Y. (2021). A Short-Term Prediction Model at the Early Stage of the COVID-19 Pandemic Based on Multisource Urban Data. IEEE Trans. Comput. Soc. Syst..

[10] Fei Z., Ryeznik Y., Sverdlov A., Tan C.W., Wong W.K. (2021). An Overview of Healthcare Data Analytics with Applications to the COVID-19 Pandemic. IEEE Trans. Big Data.

[11] Guo Z., Zhao S., Lee S.S., Hung C.T., Wong N.S., Chow T.Y., Yam C.H.K., Wang M.H., Wang J., Chong K.C. (2023). A Statistical Framework for Tracking the Time-Varying Superspreading Potential of COVID-19 Epidemic. Epidemics.

[12] Chen G.-J., Palmer J.R.B., Bartumeus F., Alba-Casals A. (2022). Modeling the Impact of Surveillance Activities Combined with Physical Distancing Interventions on COVID-19 Epidemics at a Local Level. Infect. Dis. Model..

[13] Guerrieri M., Parla G. (2022). Real-Time Social Distance Measurement and Face Mask Detection in Public Transportation Systems during the COVID-19 Pandemic and Post-Pandemic Era: Theoretical Approach and Case Study in Italy. Transp. Res. Interdiscip. Perspect..

[14] Nguyen D.C., Ding M., Pathirana P.N., Seneviratne A. (2021). Blockchain and AI-Based Solutions to Combat Coronavirus (COVID-19)-like Epidemics: A Survey. IEEE Access.

[15] Guo X., Zhang H., Kou L., Hou Y. (2022). Modeling the External, Internal, and Multi-Center Transmission of Infectious Diseases: The COVID-19 Case. J. Soc. Comput..

[16] He L., Li J., Guo Y., Sun J. (2023). Commuters’ Intention to Choose Customized Bus during COVID-19 Pandemic: Insights from a Two-Phase Comparative Analysis. Travel Behav. Soc..

[17] Lorenzo D., Mangone A., Colangeli I., Cioci P., Curini D., Vincifori V., Iannetti S. (2023). One Health System Supporting Surveillance during COVID-19 Epidemic in Abruzzo Region, Southern Italy. One Health.

[18] Ahmed I., Ahmad M., Jeon G. (2021). Social Distance Monitoring Framework Using Deep Learning Architecture to Control Infection Transmission of COVID-19 Pandemic. Sustain. Cities Soc..

[19] Gevertz J.L., Greene J.M., Sanchez-Tapia C.H., Sontag E.D. (2021). A Novel COVID-19 Epidemiological Model with Explicit Susceptible and Asymptomatic Isolation Compartments Reveals Unexpected Consequences of Timing Social Distancing. J. Theor. Biol..

[20] Abir F.F., Alyafei K., Chowdhury M.E.H., Khandakar A., Ahmed R., Hossain M.M., Mahmud S., Rahman A., Abbas T.O., Zughaier S.M. (2022). PCovNet: A Pre-symptomatic COVID-19 Detection Framework Using Deep Learning Model Using Wearables Data. Comput. Biol. Med..

[21] Youssef A.E., Alfarraj O., Alkhalaf M., Hassanein A.S. (2022). A Supervised Biosensor-Based Non-Variant Structuring Approach for Analyzing Infectious Disease Data. Measurement.

[22] Ye F., Majumder S., Jiang W., Li X., Balakrishnan N., Zhang Y., Deen M.J. (2023). A Framework for Infectious Disease Monitoring with Automated Contact Tracing—A Case Study of COVID-19. IEEE Internet Things J..

[23] Kim H., Ben-Othman J. (2022). A Successive Epidemic Prevention Infrastructure Using Mobile Robots and Smart Devices in Intelligent Public Area. IEEE Commun. Lett..

[24] Hu F., Liu J., Li L., Huang M., Yang C. (2022). IoT-Based Epidemic Monitoring via Improved Gated Recurrent Unit Model. IEEE Sens. J..

[25] Davoodi M., Ghaffari M. (2023). Learning-Based Systems for Assessing Hazard Places of Contagious Diseases and Diagnosing Patient Possibility. Expert Syst. Appl..

[26] Fu L., Yang Q., Liu Z., Liu X., Wang Z. (2022). Risk Identification of Major Infectious Disease Epidemics Based on Complex Network Theory. Int. J. Disaster Risk Reduct..

[27] Kim J., Lawson A.B., Neelon B., Korte J.E., Eberth J.M., Chowell G. (2023). Evaluation of Bayesian Spatiotemporal Infectious Disease Models for Prospective Surveillance Analysis. BMC Med. Res. Methodol..

[28] Arinik N., Interdonato R., Roche M., Teisseire M. (2023). An Evaluation Framework for Comparing Epidemic Intelligence Systems. IEEE Access.

[29] Liu R., Yang H. (2022). Spatial Tessellation of Infectious Disease Spread for Epidemic Decision Support. IEEE Robot. Autom. Lett..

[30] Rusin T.M. (2021). Forecasts of Covid-19 Evolution by Nearest Epidemic Trajectories Detection. Procedia Comput. Sci..

[31] UCF Center for Research CRCV. https://www.crcv.ucf.edu/data/ucf-qnrf/.

[32] COVID-19: Novel Coronavirus (COVID-19) Cases, Provided by JHU CSSE. https://github.com/CSSEGISandData/COVID-19.

